# *PAX7* gene polymorphism in muscular temporomandibular disorders as potentially related to muscle stem cells

**DOI:** 10.1186/s12891-021-04846-w

**Published:** 2021-11-17

**Authors:** Valquiria Quinelato, Letícia Ladeira Bonato, Alexandre Rezende Vieira, José Mauro Granjeiro, Karla Menezes, Radovan Borojevic, Priscila Ladeira Casado, Jose Albuquerque Calasans-Maia, Ricardo Tesch

**Affiliations:** 1 School of Dentistry, Universidade Federal Fluminense, Mario Santos Braga St, 28 – Center, Niterói, RJ 24020-140 Brazil; 2 University of Pittsburgh School of Dental Medicine, Pittsburgh, USA; 3 National Institute of Metrology, Quality and Technology, Rio de Janeiro, Brazil; 4 School of Medicine of Petropolis, Petropolis, Brazil

**Keywords:** Chronic pain, Temporomandibular joint dysfunction syndrome, Myalgia, Polymorphism genetic

## Abstract

**Background:**

Temporomandibular disorders (TMD) are a group of painful and debilitating disorders, involving the masticatory muscles and/or the temporomandibular joint (TMJ). Chronic TMD pain can be associated with genetic changes in the key muscle development genes.

**Objective:**

To evaluate the association between polymorphisms in the *PAX7* (paired box 7) gene and masticatory myalgia in patients with temporomandibular disorders (TMD).

**Materials and methods:**

This is a case-control study. Patients with TMD were divided into two groups: (a) presence of muscular TMD (*n* = 122) and (b) absence of muscular TMD (*n* = 49). Genomic DNA was obtained from saliva samples from all participants to allow for genotyping single nucleotide polymorphisms in *PAX7* (rs766325 and rs6659735). Over-representation of alleles was tested using chi-square or Fisher’s exact tests. Values of *p* < 0.05 were considered to be statistically significant.

**Results:**

Individuals without muscular TMD were less likely to have the *PAX7* rs6659735 GG genotype (*p* = 0.03). No associations were found for *PAX7* rs766325.

**Conclusions:**

Alterations in *PAX7* may influence muscular pathophysiology and individuals with TMD and the rs6659735 homozygous genotype (GG) are seemingly associated with muscular involvement of the disorder. No associations were found in the region rs766325.

## Background

Temporomandibular disorders (TMD) are a group of painful and debilitating disorders, involving the masticatory muscles and/or the temporomandibular joint (TMJ). Among the pain-suffering TMD patients, 50 to 70% present the diagnosis of masticatory myalgia [1], a heterogeneous pathologic condition established as a result of phenotypes, derived from a genetic mosaic, that interact with environmental factors [2].

Oral parafunction habits, such as clenching teeth during waking time, are the major clinical orofacial characteristics that predict TMD incidence [3]. During waking hours, the sustained tonic activity of the masseter muscle was significantly higher in the group of patients with orofacial pain history [4]. Experimental maximal voluntary clenching of the masseter was reported to induce nearly a five-fold reduction of oxygen saturation in the group with the high parafunction as compared to the low parafunction group, indicating that apparently healthy individuals at risk for TMD can have abnormalities in the masseter oxygenation [5]. Structural abnormalities in the masticatory muscles may be also involved, as reported in a study of two independent cohorts that compared chronic muscular TMD patients to controls [6]. Repair and regeneration of the attained tissues may be thus required, involving mobilization and activation of the resident muscle progenitor cells.

Recent studies on muscle functions have proposed that maintenance of the TMD chronic pain can also depend upon the patient’s genetic profile and the presence of locally produced or circulating tissue factors, which may result in chronic inflammation of muscles [7]. There is direct evidence for increased levels of the pro-inflammatory cytokines in the skeletal muscle performing low force tasks, suggesting that chronic repetitive activities could initiate a chronic inflammatory response in the working muscles [8]. While acute inflammation can result in muscle edema, a chronic inflammation can result in muscle destruction [9]. Hypo-oxygenation of muscles suffering chronic inflammation may increase tissue damage, predisposing the muscle to further injury, thus minimizing the probability of adequate repair [10].

While many cells are involved in chronic inflammation, macrophages, through their association with skeletal muscle stem cell, satellite cell (SC) activation, may be one of the major players in the control of injury and impaired muscle regeneration. An imbalance of M1 and M2 macrophages may lead to impaired SC activation in skeletal muscle regeneration after damage. Therefore, strategies reverting chronic inflammation into a pro-regenerative reaction appear to be appropriate. Recent studies suggest that muscular manual acupuncture produces a phenotypic switch in macrophages and increases IL-10 concentrations in muscle, reducing pain and inflammation [11]. Conversely, the manual massage facilitates membrane permeability, which can be associated with an increase in satellite cell number [12].

Satellite cells (SC), the stem cells of skeletal muscle, are required for skeletal muscle recovery. Their functionality is modulated by intrinsic signaling pathways as well as by their interactions with the stem cell niche [13]. Following muscle injury, SC are activated, proliferate extensively, and ultimately differentiate into myoblasts, fusing with existing fibers or other SC to form new myofibers [14]. In healthy individuals, expansion of the SC pool was identified in the cervical muscles after heavy resistance training [15], as well as low-force muscle loads [16]. Repetitive muscle overloading may also result in increased inflammatory and myogenic activity, and painful muscle fibers may require higher numbers of SC than muscle fibers not exposed to this recruitment. Craniofacial muscles have a higher proportion of SC as compared to other skeletal muscles, which may increase their capacity for regeneration in response to tissue damage under normal conditions [17].

At the lesion site, growth factors are required for activating SC. Expression of the transcription factor *PAX7* (paired box 7) is fundamental to the control of muscle cell differentiation [18, 19]. Quiescent resident SC expressing *PAX7* can be induced to migrate into the lesion site, then becoming proliferative and expressing, in addition to *PAX7*, the myogenic regulatory factor, *MyoD* (myogenic differentiation). The final differentiation of these SC into myoblasts is marked by reduction of *PAX7* that is substituted progressively by an increased *MyoD* expression. The fusion of differentiated myoblasts generates new myofibrils or repairs the damaged ones [20].

Reduced *MyoD* expression and the maintenance of a high *PAX7* expression is required for the maintenance and self-renewal of SC [21]. When the SC population is activated, its fraction undergoes a terminal differentiation while the other one remains in the pool of quiescent satellite cells, which can be induced again to proliferate, to be activated and to participate in regeneration and control of inflammation. The size of this resident pool of SC and its capacity to attend the local demands may control the response to injury in qualitative and quantitative terms, and this is known to be regulated by the *PAX7* activity [22].

These data were the basis of the here-proposed hypothesis that polymorphisms in the *PAX7* gene could modulate the activity of this gene to influence the regenerative role played by SC in the muscular environment. We believe that this genetic pattern may influence the development and chronic persistence of muscle disorders, including the masticatory ones. However, more recent studies indicate that the involved mechanisms are almost exclusively attributed to facilitation, peripheral and central, in processing pain. As far as we are aware, the presence of polymorphisms in genes associated with muscle tissue embryogenesis and tissue regeneration processes have not yet been investigated with respect to the development of masticatory myalgia.

The objective of the present study was to test the hypothesis that polymorphisms in the *PAX7* gene are associated with the presence of masticatory myalgia in patients with TMD.

## Materials and methods

The present clinical study is descriptive and cross-sectional, being evaluated and approved by the Research Ethics Committee of the Antônio Pedro University Hospital of the Fluminense Federal University on September 25, 2016 (protocol number 1.744.837). All of the subjects consented freely to participate in this project. Informed consent forms were received and signed by the participants before the research was conducted. All methods were carried out in accordance with relevant guidelines and regulations. The design development of this study followed the recommendations of *Strengthening the Reporting of Observational Studies in Epidemiology* (STROBE) [23].

The study included patients attended at the Fluminense Federal University (18 to 65 years) as previously described [24]. They were randomly selected over a period of 6 months. Exclusion criteria were the history of macro trauma and/or surgery in the temporomandibular region, diagnosis of rheumatoid arthritis, fibromyalgia, or other types of systemic musculoskeletal diseases, and/or previous treatment for TMD. The inclusion criteria were the presence of one or multiple TMD diagnoses.

All participants were clinically examined by the same evaluator (co-author L.L.B.), using the Research Diagnostic Criteria for Temporomandibular Disorders (RDC/TMD) – Axis I [25], validated for the physical diagnosis of TMD, and enabling classification of participants in one of the following diagnostic subgroups: (0) absence of TMD; (1) myofascial pain; (2) changes in the position of the articular disc; (3) painful and/or degenerative TMJ conditions. This process occurred in a non-mutually exclusive manner, allowing each participant to belong to more than one diagnostic sub-group. Participants were then grouped into three groups: (a) control (absence of TMD); (b) with muscular TMD; (c) without muscular TMD.

The decision of including patients with absence of TMD as a control group assumed that they were probably not strongly subjected to the common risk factors for the development of these disorders, such as muscular and joint overloading by oral parafunctional habits. This is not a study of TMD risk, but a study of muscular involvement predisposition in patients with TMD.

Genomic DNA was obtained from saliva samples from all participants, as previously described [26]. The concentration and purity of the DNA were analyzed using the NanoDrop® spectrophotometer (Thermo Scientific, Wilmington, DE, USA). All samples had to present an A260 nm / A280 nm ratio higher than 1.9.

Two polymorphisms of a single nucleotide (SNP) in the *PAX7* gene (rs766325, rs6659735) were selected, considering the linkage disequilibrium and gene structure relationships. These SNPs were previously identified and included in the database of the National Center for Biotechnology Information (http://www.ncbi.nlm.nih.gov/SNP/), with the lowest allele frequency having to be > 012. All procedures followed the recommendations of the Strengthening the Reporting of Genetic Association Studies (STREGA) [27].

**Fig. 1:**
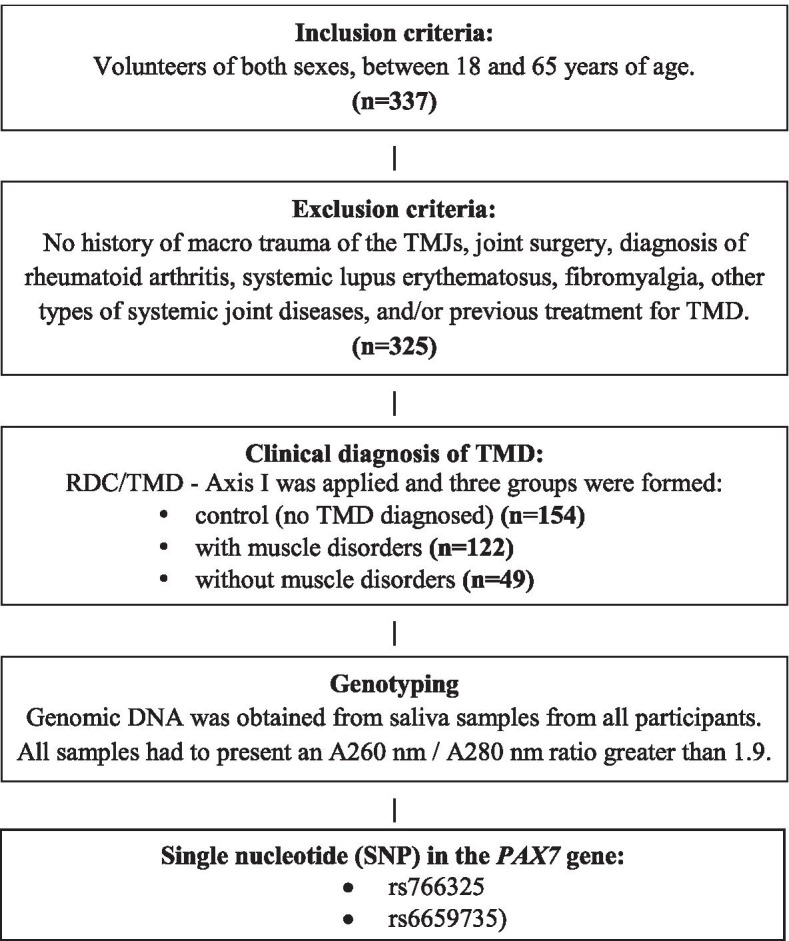
Flow chart showing the inclusion of patients, groups and SNP in this study

For a better understanding, the methodology steps are illustrated in Fig. 1.

Data processing and statistical analyses were done using STATA 12.0 (Stata Corp., College Station, TX, USA). The sample size included the spontaneous demand of patients over 6 months, respecting the inclusion criteria. Using data from a previous study under conditions of a 95% confidence interval and 90% power of test, the sample was calculated considering a 40% prevalence of TMD [28]. Considering a loss of 5%, the estimated number to produce adequate statistical power was 200 individuals. Since the main inclusion criterion of the sample was the presence of TMD regardless of the type, we were able to obtain enough individuals. However, when the sample was stratified into different TMD subgroups, the number of individuals in each group was below the size of the calculated sample, therefore the differences between the groups were considered to be significant only after the statistical analysis using Fisher’s exact test with an alpha of 0.05.

Differences between groups in the frequency of genotypes and alleles were analyzed using the chi-square test after assembly for the Hardy-Weinberg equilibrium. Statistical differences between groups were calculated using the chi-square or Fisher’s exact tests. To calculate linkage disequilibrium and diplotypes, the ARLEQUIN software program was used (http://cmpg.unibe.ch/software/arlequin3/). Values of *p* < 0.05 were considered to be statistically significant, and the risks associated with individual alleles and genotypes were calculated as odds ratio (OR) with a 95% confidence interval (CI).

## Results

From the 337 volunteers evaluated over the six-month period, after the adoption of the exclusion criteria, 325 were included in the study. A total of 154 (47.3%) did not have a TMD diagnosis, 122 (37.5%) patients had muscular TMD while 49 (15%) had only non-muscular TMD.

The group with muscular TMD consisted of 85 women and 37 men, and the group without muscular TMD consisted of 32 women and 17 men. The mean age found was 45.1 ± 12.3 years. There was no significant difference between gender and the risk of developing muscular (*p* = 0.17) or other type disorders (*p* = 0.71). The characteristics of the two polymorphisms studied in the *PAX7* gene are presented in Table 1.

**Table 1:** Characteristics of the PAX7 gene polymorphisms studied

Gene symbol	Gene name	SNP	Chromosome	Base pair position*	SNP type	Base change^a^		MAF^b^
						Major	Minor	
*PAX7*	paired box 7	rs766325	1	18,629,964	Upstream	A	G	0.48
		rs6659735		18,657,203	Intragenic	A	G	0.26

*SNP* Single nucleotide polymorphism. / ^a^ Base change according to Applied Biosystems. / ^b^ MAF: minor allele frequency according to GenBank

The integrity rate of the genomic DNA was 92.3%, with a high correspondence rate between replicate samples, indicating a highlevel of confidence in accurate and unbiased genotyping. The frequencies of the genotypes and alleles are shown in Table 2.

**Table 2:** Distribution of the genotypes and alleles of the PAX7 gene

Gene	SNP	Genotypes	Control ***N*** = 154	With Muscular TMD ***N*** = 122	Without Muscular TMD ***N*** = 49	***P***-value* (OR;CI)	
						***Control x With Muscular TMD***	***With Muscular TMD x Without Muscular TMD***
***PAX7***	rs766325	AA-AG-GG	40–53-52	32–34-42	16–14-17	0.7	0.86
		AG + GG	105	76	31	0.82 (0.9 (0.5–1.5)	0.71 (0.8 (0.3–1.6)
		A	133	98	46	0.49 (0.9 (0.6–1.3)	0.64 (1.1 (0.7–1.8)
		G	157	118	48		
	rs6659735	AA-AG-GG	73–58-11	64–42-8	32–14-0	0.75	**0.03**
		AG + GG	69	50	14	0.53 (0.82 (0.5–1.3)	0.16 (0.5 (0.2–1.1)
		A	204	170	78	0.55 (1.1 (0.7–1.7)	0.06 (1.9 (1–3.6)
		G	80	58	14		

No significant associations were found in the gene haplotype analysis (Table 3)

**Table 3:** Analysis of diplotypes

Gene	Diplotype	Frequency			***P***-value	
		Control ***N*** = 154	With Muscular TMD ***N*** = 122	Without Muscular TMD ***N*** = 49	***Control x*** With Muscular TMD	With Muscular TMD ***x*** Without Muscular TMD
***PAX7*– Ch1 (rs766325; rs6659735)**	AA	0.43	0.43	0.47	–	–
	GA	0.27	0.31	0.36	0.53	0.5
	GG	0.26	0.23	0.14	0.58	0.08
	AG	0.02	0.01	**0.01**	0.76	0.75

The analysis of the *PAX7* rs6659735 marker showed a lower-representation of the GG genotype among individuals with non-muscular TMD, when compared to the muscular TMD group (*p* = 0.03) (Table 2).

## Discussion

The present clinical study analyzed the possible associations of the polymorphisms observed in the gene coding *PAX7* transcription-factor with the presence and with clinical aspects of the muscular TMD. The results showed that the patients not bearing the GG genotype of rs6659735 were apparently protected from the muscular involvement in the TMD disorders.

Being concerned with multiple testing, we avoided applying the strict Bonferroni correction, increasing the Type II error. Using this correction, we would have lowered the alpha to 0.025 (0.05/2). It has been previously shown that the known associations are missed when correction for multiple testing is implemented [29].

The *PAX7* rs766325 SNP was associated with craniofacial skeletal variations in patients with malocclusions [30]. This polymorphism was described in two studies, which investigated their relationship with palate and lip clefts [31, 32]. The SNP is located upstream of the gene and does not influence formation of the protein. Conversely, the less common homozygous genotype of rs6659735 showed a suggestive effect on the muscular TMD.

The major functions of the *PAX7* gene in skeletal muscle are control of the basal reserve population of resident muscular SC, and of their mobilization when attending to a need for regeneration after injury, as well as in the case of muscular volume increase in response to sustained efforts. These SC reside in the sub-fascial and perivascular niches within muscles, remaining in the phase zero of the cell cycle. They can be activated rapidly in response to injury [33]. In this case, SC re-enter the cell cycle upon activation, migrate from their niche into the regenerating region and engage in formation and maintenance through a controlled renewal of the proliferating myoblast pool. These cells are subsequently engaged in their terminal differentiation into myocytes, which merge into preexisting damaged muscle fibers or fuse with other cells to generate new muscle fibers [21]. The cell regeneration requires the progressive interruption of the *PAX7* gene expression and its substitution by expression of *MyoD*, which is required for the myocyte differentiation.

Alternatively, depending upon intensity and duration of injuries, a portion of activated SC can re-enter into quiescence, return to their niche and again express *PAX7*. This is a self-renewal of the muscular SC pool, and it is required for a long-term maintenance of the basal SC pool that remains available for the new waves of a regenerative supply of myoblasts, when required. The *PAX7* gene activity is thus in charge of the delicate controls within the cells that supply new myoblasts that engage into the myocyte terminal differentiation, and resident cells that remain available for subsequent waves of regenerative production of new myoblasts [34]. In chronic or repetitive aggressions of muscle tissue, the equilibrated *PAX7* activity has to grant both the immediate and efficient tissue regeneration and the long-term maintenance of the resident SC pool providing regeneration for the future demands.

Our study does not supply information on the properties and activities of the *PAX7* gene in the context of the GG genotype polymorphism. Since it has clearly a long-term effect on the muscular involvement in the TMD disorder, we may raise the hypotheses of either decreased or increased responses of the *PAX7* gene activity to the tissue environment in the context of TMD, as compared to controls.

In the former case, the decreased operational activity of *PAX7* in muscular SC populations, before or along their engagement into myoblast differentiation, will potentially lead to an increased or accelerated supply of *PAX7*-negative and *MyoD*-positive myocytes to be engaged in the muscle fiber repair. In acute lesions this can lead to a more rapid and efficient tissue response and regeneration. In chronic and repetitive lesions, we can expect an association with a low reposition or exhaustion of the resident SC pool, which requires a high level of *PAX7* activity. Conversely, in the case of increased total activity of *PAX7* within the muscular SC population, we can expect a somewhat delayed substitution of *PAX7* by the *MyoD* expression, with a potential delay in myocyte differentiation. However, this can lead to a permanent increase of the resident SC pool, with an increased long-term capacity to respond to chronic or repetitive injuries, and an increased capacity for repair and regeneration.

The similar rationale may be applied to the question of the relationship with the inflammatory processes that are associated with TMD that can be one of the causes of the associated pain. The *PAX-7* activity has limited direct relationship with mobilization and activity of inflammatory cell populations. However, muscle SC can interfere with maturing macrophages by insulin-like growth factor-2, inducing M1-M2 macrophage conversion and an overall anti-inflammatory protection [35]. A more robust resident SC population can thus have an increased basal anti-inflammatory capacity. The *PAX-7* controls of the resident muscle SC population can potentially influence a broad set of tissue reactions to stress. The proposed hypotheses should be addressed in experimental in vitro and in vivo models and are the object of ongoing studies.

## Conclusion

Alterations in the *PAX7* may influence muscular pathophysiology since individuals with TMD and the rs6659735 homozygous genotype (GG) seem to be associated with muscular involvement of the disorder. This result leads to the proposal of an unexplored stem cell related genetic predisposition to muscular TMD.

## Data Availability

The datasets generated or analyzed during the current study are available from the corresponding author upon reasonable request; SNPs were previously identified and included in the database of the National Center for Biotechnology Information (http://www.ncbi.nlm.nih.gov/SNP/), with the lowest allele frequency having to be > 012.

## Abbreviations

*PAX7*: Transcription factor *PAX7* (paired box 7)
TMD: Temporomandibular disorders
TMJ: Temporomandibular joint
SC: Satellite cells
SNP: Single nucleotide polymorphism
